# MARINE AND COASTAL SCIENCE: Ocean Currents Key to Methylmercury in North Pacific

**DOI:** 10.1289/ehp.117-a345

**Published:** 2009-08

**Authors:** Carol Potera

**Affiliations:** Montana-based **Carol Potera** has written for *EHP* since 1996. She also writes for *Microbe*, *Genetic Engineering News*, and the *American Journal of Nursing*

Mercury emitted from Asian coal-fired power plants travels long distances via ocean currents and raises mercury levels in the North Pacific Ocean, report researchers who measured mercury at 16 ocean sites between Hawaii and Alaska. Their findings may explain why mercury levels are increasing in waters of the eastern North Pacific when no local source of mercury is apparent and suggest, moreover, that fish mercury levels may respond in kind. By 2050, wrote Elsie M. Sunderland and colleagues, mercury levels in the North Pacific could double relative to 1995 levels if anthropogenic emissions remain at their present levels, including coal use in Asia. The study, reported in the 1 May 2009 issue of *Global Biogeochemical Cycles*, is the first to document methylmercury formation in the Pacific Ocean.

About 24% of U.S. mercury intake comes from Pacific tuna, according to research published in the February 2007 issue of *EHP* by Sunderland, now a research associate at Harvard University. Mercury is readily transformed into methylmercury, a potent, persistent neurotoxicant. Methylmercury impairs neurodevelopment in children and raises the risk for cardiovascular disease in adults. Methylmercury, the only form of mercury that bioaccumulates in fish, accounts for more than 95% of the total mercury in predatory species such as tuna, says Sundlerland.

In water samples collected by the researchers, methylated mercury species were particularly elevated at intermediate depths (200–700 m) but not in surface or deeper waters. Moreover, mercury levels had increased up to 30% since 1995, but only in intermediate-depth samples. “That left us scratching our heads,” says coauthor David Krabbenhoft, a research hydrologist with the U.S. Geological Survey. The conventional thinking has been that ocean mercury comes from direct atmospheric deposition onto the sea surface or from deep underwater volcanoes, yet Krabbenhoft and colleagues detected little mercury in surface and deep waters.

The mystery was solved by atmospheric and ocean circulation models created by Sunderland and coauthor Sarah Strode of the University of Washington in Seattle. The data pointed to emissions from the heavily populated coast of Asia as the source of mercury. The toxic metal was deposited in the western Pacific, then ocean currents carried it to the eastern North Pacific study sites within two years, according to the model.

The authors found that the majority of mercury settles to mid-level waters because it sticks to surface algae that die and sink. When underwater bacteria degrade the algae in the presence of mercury, methylmercury is produced. The team confirmed that the highest methylmercury levels at intermediate depths coincided with high organic carbon utilization and oxygen depletion, markers for microbial decomposition. Larger species of tuna also live in the 200- to 1,000-m mesopelagic zone, according to the Food and Agriculture Organization of the United Nations.

A few days after publication, an independent team of French scientists released nearly identical results for the Mediterranean Sea in the May 2009 issue of *Limnology and Oceanography*. They also found maximum methylmercury concentrations at intermediate depths produced by bacterial breakdown of organic matter. Vincent St. Louis, a professor of biological sciences at the University of Alberta, and colleagues reported similar findings for Arctic waters in the 15 November 2008 issue of *Environmental Science & Technology*. Most aquatic mercury research has focused on lakes, and “people are just starting to understand what’s going on in the ocean,” says St. Louis. The new findings “help us to understand where methylmercury is produced in oceans,” he says.

The studies also shed light on the environmental riddle of why fish caught in some waters have high mercury levels despite the lack of a local source. Alaska’s Fish Monitoring Program evaluates marine mercury levels statewide. State Veterinarian Robert Gerlach has observed up to a 150% rise in mercury levels in halibut from the Bering Sea compared with 1976 levels, but halibut from the southeastern Gulf of Alaska have shown only small increases in mercury. “My assumption is that the increase is due primarily to atmospheric deposition from coal-fired power plants in Asia and long-range transport on ocean currents,” says Gerlach. Sunderland’s models suggest that Asian ocean currents travel northeast over the Bering Sea but bypass the Gulf of Alaska, supporting Gerlach’s idea.

## Figures and Tables

**Figure:**
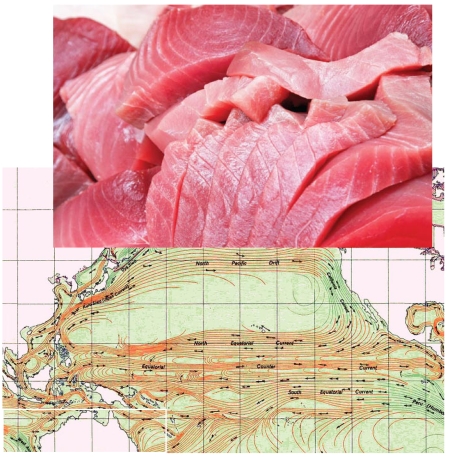
Pacific currents play a key role in how methylmercury winds up in fish.

